# Microsaccadic suppression of peripheral perceptual detection performance as a function of foveated visual image appearance

**DOI:** 10.1167/jov.24.11.3

**Published:** 2024-10-04

**Authors:** Julia Greilich, Matthias P. Baumann, Ziad M. Hafed

**Affiliations:** 1 Werner Reichardt Centre for Integrative Neuroscience, University of Tübingen, Tübingen, Germany; 2 Hertie Institute for Clinical Brain Research, University of Tübingen, Tübingen, Germany

**Keywords:** microsaccades, saccadic suppression, foveal vision, spatial frequency, perisaccadic perception

## Abstract

Microsaccades are known to be associated with a deficit in perceptual detection performance for brief probe flashes presented in their temporal vicinity. However, it is still not clear how such a deficit might depend on the visual environment across which microsaccades are generated. Here, and motivated by studies demonstrating an interaction between visual background image appearance and perceptual suppression strength associated with large saccades, we probed peripheral perceptual detection performance of human subjects while they generated microsaccades over three different visual backgrounds. Subjects fixated near the center of a low spatial frequency grating, a high spatial frequency grating, or a small white fixation spot over an otherwise gray background. When a computer process detected a microsaccade, it presented a brief peripheral probe flash at one of four locations (over a uniform gray background) and at different times. After collecting full psychometric curves, we found that both perceptual detection thresholds and slopes of psychometric curves were impaired for peripheral flashes in the immediate temporal vicinity of microsaccades, and they recovered with later flash times. Importantly, the threshold elevations, but not the psychometric slope reductions, were stronger for the white fixation spot than for either of the two gratings. Thus, like with larger saccades, microsaccadic suppression strength can show a certain degree of image dependence. However, unlike with larger saccades, stronger microsaccadic suppression did not occur with low spatial frequency textures. This observation might reflect the different spatiotemporal retinal transients associated with the small microsaccades in our study versus larger saccades.

## Introduction

Microsaccades are small saccades that occur periodically during attempted gaze fixation (Rolfs, 2009). These eye movements are similar to larger scanning saccades in both kinematics and underlying neurophysiological mechanisms (Hafed, 2011; Hafed, Goffart, & Krauzlis, 2009; Hafed & Krauzlis, 2012; Zuber, Stark, & Cook, 1965). Moreover, microsaccades aid in optimizing gaze position during foveal visually guided behavior (Bellet, Chen, & Hafed, 2017; Ko, Poletti, & Rucci, 2010; Poletti, Listorti, & Rucci, 2013; Shelchkova, Tang, & Poletti, 2019; Tian, Yoshida, & Hafed, 2016, 2018), just like larger saccades allow targeting new scene locations for detailed visual analysis. Microsaccades may thus be thought of as scanning eye movements but on the small scale of foveal vision (Hafed, Chen, Tian, Baumann, & Zhang, 2021).

Because of the similarity between microsaccades and saccades, it is expected that both types of eye movements may similarly affect visual processing. Indeed, some of the earliest studies on the well-known phenomenon of perceptual saccadic suppression were first conducted with microsaccades (Beeler, 1967; Zuber & Stark, 1966). In this phenomenon, the detection of brief perisaccadic or perimicrosaccadic visual stimulus onsets is strongly impaired. Later work characterized the neural correlates of this phenomenon, again with microsaccades. Specifically, and using a similar approach to that used in psychophysical studies (i.e., by presenting visual onsets around the time of microsaccade onset), it was found that visual neural sensitivity in multiple brain areas can be suppressed for perimicrosaccadic stimulus events (Chen & Hafed, 2017; Chen, Ignashchenkova, Thier, & Hafed, 2015; Hafed & Krauzlis, 2010). These studies complemented other studies demonstrating an influence of both saccades and microsaccades on ongoing visual neural activity in the absence of sudden stimulus onsets (Bosman, Womelsdorf, Desimone, & Fries, 2009; Crowder, Price, Mustari, & Ibbotson, 2009; Herrington et al., 2009; Kagan, Gur, & Snodderly, 2008; Leopold & Logothetis, 1998; MacEvoy, Hanks, & Paradiso, 2008; Reppas, Usrey, & Reid, 2002; Sylvester, Haynes, & Rees, 2005; Thiele, Henning, Kubischik, & Hoffmann, 2002; Wurtz, 1968, Wurtz, 1969a, Wurtz, 1969b).

With large saccades, the phenomenon of perceptual saccadic suppression has been extensively studied for several decades. While the underlying mechanisms for this phenomenon may not be fully elucidated yet, increasing evidence suggests that saccadic suppression arises through a potential interaction between the movement command for saccade generation itself and the viewed visual image properties (Baumann, Idrees, Munch, & Hafed, 2021; Braun, Schutz, & Gegenfurtner, 2017; Bremmer, Kubischik, Hoffmann, & Krekelberg, 2009; Brooks, Impelman, & Lum, 1981; Duffy & Lombroso, 1968; Gremmler & Lappe, 2017; Idrees, Baumann, Franke, Munch, & Hafed, 2020; Mackay, 1970; Maij, Matziridi, Smeets, & Brenner, 2012; Matin, Clymer, & Matin, 1972; Mitrani, Mateeff, & Yakimoff, 1971; Mitrani, Yakimoff, & Mateeff, 1973; Riggs & Manning, 1982; Wurtz, 2008). In fact, the dependence of perceptual saccadic suppression on the properties of the viewed visual image already starts in the retina, the very first visual processing stage after the eye optics (Idrees et al., 2020; Idrees et al., 2022). Interestingly, if motor commands of the superior colliculus do contribute to saccadic suppression via ascending corollary discharge projections (Berman, Cavanaugh, McAlonan, & Wurtz, 2017; Berman & Wurtz, 2011; Isa & Hall, 2009; Lee et al., 2007; Phongphanphanee et al., 2011), then an interaction between saccade movement commands and underlying visual image properties for saccadic suppression becomes almost inevitable, especially given the discovery of visual sensory tuning within the superior colliculus motor bursts themselves (Baumann, Bogadhi, Denninger, & Hafed, 2023; Zhang, Malevich, Baumann, & Hafed, 2022).

Having said that, most prior work on dependencies of perceptual saccadic suppression on visual image appearance has focused on large saccades (but for a notable exception, see Scholes, McGraw, & Roach, 2018). Thus, here, we aimed to document potential changes in the properties of microsaccadic suppression of perceptual detection performance as a function of what image was being fixated when a microsaccade was generated. Using an approach similar to that we used recently (Idrees et al., 2020), we measured peripheral perceptual detection thresholds and sensitivity (slopes of psychometric curves) when microsaccades were generated over a stable background image. We found that both detection thresholds and sensitivity were generally impaired perimicrosaccadically but that only threshold elevations (and not sensitivity reductions) showed a dependence on the visual appearance of the image that was foveated. Moreover, the visual dependence of threshold elevations was decidedly different from that expected from our earlier results with larger saccades (Idrees et al., 2020), possibly reflecting the largely different spatiotemporal scales with which microsaccades and saccades modulate retinal image statistics (Mostofi et al., 2020). Our results complement earlier investigations, in both humans and monkeys, of the diverse relationships between microsaccade generation and the visual processing of different spatial frequencies (Chen & Hafed, 2017; Hass & Horwitz, 2011; Intoy, Mostofi, & Rucci, 2021; Scholes et al., 2018), and they motivate neurophysiological and computational investigations of these relationships.

## Methods

### Subjects and ethical approvals

We recruited 12 human subjects for this study. Of these, seven were female, and five were male. The subjects were aged 21 to 42 years, and each took part in three experimental sessions. The first and last sessions included 690 trials each, and the second session included 675 trials. Two subjects had too few baseline trials (defined explicitly in more detail below), so they each performed an additional session of 600 trials. The subjects individually took up to three short breaks during each session. All subjects consented to the experiment, and the procedures were approved by ethical committees of the Medical Faculty of the University of Tübingen. The subjects, who were naive to the purposes of the study, were also compensated financially for their time.

### Laboratory setup

All experiments were performed in the same setup as that used for recent studies (Baumann et al., 2021; Idrees et al., 2020; Idrees et al., 2022). In brief, the subjects sat comfortably 57 cm in front of a CRT display spanning approximately ± 17 deg horizontally and ± 13 deg vertically. The display had a refresh rate of 85 Hz and a pixel resolution of 41 pixels/deg. The display was also calibrated and linearized for luminance, as we used a similar procedure of collecting psychometric curves as in our earlier studies on perceptual thresholds (Baumann et al., 2021; Idrees et al., 2020). The room was otherwise dark.

We tracked eye movements using a video-based eye tracker (EyeLink 1000; SR Research), which was desk-mounted. This required stabilizing the head position, which we did using a custom-built device involving a chin rest, a forehead rest, constraints around the temple of the head, and a head band wrapped behind the head (Hafed, 2013).

We controlled the experiments using the Psychophysics Toolbox (Brainard, 1997; Kleiner, Brainard, & Pelli, 2007; Pelli, 1997) and Eyelink Toolbox (Cornelissen, Peters, & Palmer, 2002).

### Stimuli

**Figure 1.**
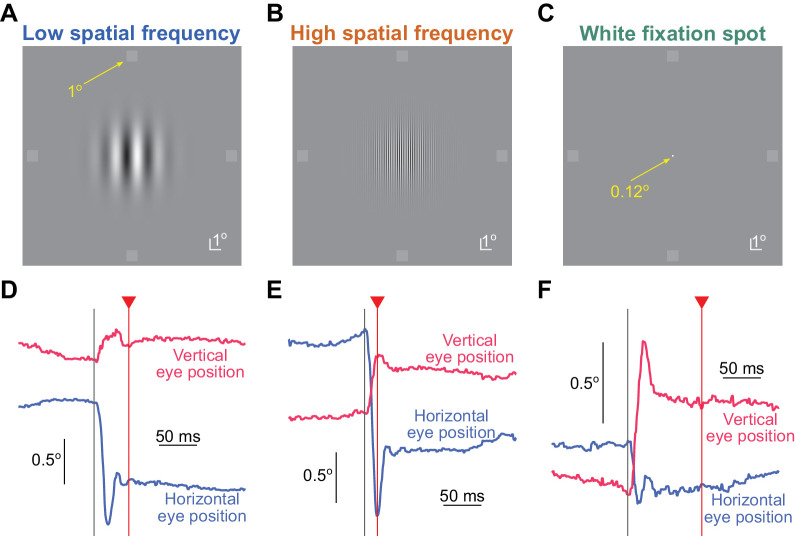
Experimental paradigm. (**A**–**C**) The images that were fixated in this study (Methods), along with the possible brief probe flash locations (small dim squares in the periphery). In every trial, the subjects were instructed to fixate near the center of the image. At a variable time from online microsaccade detection, a brief probe flash was presented at one of four peripheral locations (right, left, up, or down from the image center; we show the four probes here simultaneously only for illustration purposes because only one flash was presented per trial). The subjects had to indicate where they saw the brief probe flash. The luminance of the probe flash varied from trial to trial, in order to collect full psychometric curves. (**D**–**F**) Example eye position traces and probe flash onset times (vertical red lines) from one example subject (S05) in each of the image conditions shown in **A** to **C**. An upward deflection in the shown eye position traces indicates a rightward gaze shift for horizontal eye position and an upward gaze shift for vertical eye position. Our goal was to assess perceptual detection performance as a function of the time of flash onset relative to microsaccade onset and also as a function of the different underlying foveated images. Note how the probe flash time was variable relative to microsaccade onset across different trials.

We asked the subjects to always maintain fixation on an image that was presented at the center of the display in every trial. Across trials, three different images were possible (Figures 1A–C). The first two were vertical Gabor gratings, and the last was a small, white fixation spot. The white fixation spot was a square of 0.12 deg and 94.91 cd/m^2^ luminance. The Gabor gratings could have a spatial frequency of either 0.5 cycles/deg (referred to as the low spatial frequency grating) or 5 cycles/deg (referred to as the high spatial frequency grating). These Gabors had a σ parameter of 1.75 deg, and they thus visually spanned approximately ± 6 deg horizontally and vertically on the display. The underlying sine wave luminance of each grating had a contrast of 100%, and we randomly picked one phase on every trial (from eight possible phases equally spaced between 0 and 2π). The gray background luminance was 22.15 cd/m^2^.

For the Gabor gratings, we chose 0.5 cycles/deg and 5 cycles/deg, in particular, in order to fulfill three equally important desirables for our study. First, we wanted to have sufficient visibility of the images at the two chosen spatial frequencies, and this was the case for both 0.5 and 5 cycles/deg (especially at the high contrast levels that we used). Thus, the two grating images were clearly visible to the subjects, despite their different underlying spatial frequency patterns. Second, and at the same time, we additionally wanted to have sufficient image differences between the low and high spatial frequencies of our paradigm, especially with respect to the expected spatiotemporal retinal image modulations caused by microsaccades (Hafed, Chen, & Khademi, 2022; Intoy et al., 2021; Khademi, Chen, & Hafed, 2020; Mostofi et al., 2020). For example, we expected that the small microsaccade sizes that we studied (see Results) might cause smaller luminance modulations on the retina when made across the low spatial frequency grating than across the high spatial frequency grating. Finally, we also wanted to have a range of spatial frequencies that were in line with those used in our earlier studies (Baumann et al., 2023; Idrees et al., 2020), so that we could compare and contrast the effects of small and large saccades on similar image conditions. This is important to do since we always perform combinations of small and large saccades in naturalistic gaze behavior (MacEvoy et al., 2008).

**Figure 2.**
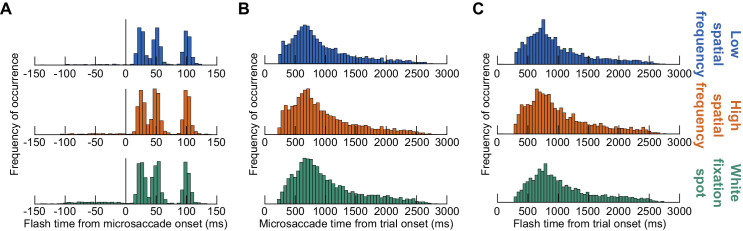
Better synchrony of probe flash times to microsaccade onset than to image (trial) onset. (**A**) Likelihood of a probe flash across all experiments as a function of time from microsaccade onset (for all trials accepted into the analyses). Each row shows an image condition. As can be seen, there were three clear and punctate peaks after microsaccade onset, consistent with our gaze-contingent triggering algorithm (Methods). (**B**) Likelihood of microsaccade onset from trial (image) onset in our experiments. Each row represents an image condition. Note that there was a much wider time range on the x-axis than in A. Thus, the relationship of probe flash time from microsaccade onset (**A**) was maintained irrespective of whether the microsaccade itself happened early in a trial or after more than 2 seconds from trial onset. Otherwise, the histograms of A would have looked much more blurred. (**C**) Consistent with this, because flash times were synchronized with microsaccade onset (**A**), they happened over a very wide range of times from trial onset, just like the microsaccades themselves. We also saw no correlation between flash time from microsaccade onset and flash time from trial onset, suggesting that our triggering of probe flash times relative to microsaccade onsets was stationary in time, and it persisted whether a microsaccade occurred early or late in a trial. Note that **B** and **C** show distributions from all microsaccade directions, but the underlying distributions were the same even for the predominantly horizontal microsaccades used in the rest of our analyses for this study (see Figure 3 and Methods).

To probe perceptual thresholds, we also presented brief flash stimuli, which were squares of 1 deg size (each trial was associated with only a single probe flash presentation, as described in more detail below). These probe stimuli were presented at 9.1 deg from the screen center either horizontally or vertically, and their luminance varied across trials. This allowed us to collect full psychometric curves of detection performance. Specifically, each flash probe had a luminance increment above the background screen luminance of 2, 4, 5, 6, 8, 10, 11, or 14 computer register steps, with each step causing an actual luminance increase of 0.56 cd/m^2^ (which we measured after display calibration). The range of luminance steps used in any given condition depended on the time at which we presented the probe flash relative to a detected microsaccade (see details below). Specifically, our experiments involved gaze-contingent microsaccade detection (Baumann et al., 2021; Chen & Hafed, 2013; Idrees et al., 2020), and we expected (Beeler, 1967; Zuber & Stark, 1966) higher perceptual thresholds for flashes very close to microsaccade onset than for later flashes. Thus, we used luminance increment steps of 2, 5, 8, 11, and 14 for the trials with higher expected perceptual thresholds, and we used 2, 4, 6, 8, and 10 increment steps for the trials with flashes farther away from microsaccades. In post hoc analyses (see below), we redetected microsaccades and reclassified flash times (Figures 1D–F, 2A), and we used all available luminance increments of a given trial type for fitting the psychometric curves.

### Experimental procedures

Each session took approximately 50 to 60 minutes. In each trial, a central image first appeared (low spatial frequency grating, high spatial frequency grating, or white fixation spot). The subjects were asked to maintain fixation on the image. For the white fixation spot, this was easy because it was the only visible item on the display, and it was very small. For the gratings, we instructed the subjects to look somewhere near the middle of the image. This allowed equalizing the eccentricity of the probe flashes across their four possible locations (also see Results). After 250 to 750 ms from image onset, we started a computer process of monitoring eye positions in real time (Baumann et al., 2021; Idrees et al., 2020). If a microsaccade was detected after a randomized time between 1,600 ms and 2,000 ms, we presented a probe flash for only one display frame at one of four possible locations (as described above). Moreover, the probe flash was triggered at 0, 25, or 75 ms after online eye movement detection. If no microsaccade was detected by our timeout period, we presented the probe flash anyway at one of the four locations. Later, in post hoc analyses, we checked for the time of the nearest microsaccade to probe flash onset in this case, and we classified the trial according to our standard time course analyses (see more data analysis details below). After the probe flash, the computer waited for the subject to press one of four buttons, indicating the perceived flash location (right, left, up, or down). If no button was pressed after 1 second, a text message appeared on the display asking the subject to respond (and guess the flash location if necessary).

Our process for online microsaccadic eye movement detection was described earlier (Chen & Hafed, 2013) and successfully used for both microsaccades (Chen & Hafed, 2013) and larger saccades (Baumann et al., 2021; Idrees et al., 2020). Briefly, in every millisecond, we collected a running series of the latest 5 ms of eye position samples. Within each collection, we estimated the rate of change of eye position by fitting a line to the collected samples. To reduce effects of noise, we then took the median of the latest three slope measurements and flagged a microsaccade occurrence if the value of the slope was larger than a user-adjusted threshold. Note that this procedure necessarily delayed our estimate of microsaccade onset. This is why we redetected all microsaccades in later offline analyses after data collection, and we then recalculated probe flash times to actual microsaccade onset times (Figures 1D–F, 2A). Also note that with this approach, we did not systematically measure premicrosaccadic perceptual performance. This was the case because it would have required excessively more trials: Without experimental control on microsaccade onset time, collecting premicrosaccadic performance would entail presenting flashes at random times and then collecting enough trials to catch ones in which the flashes occurred premicrosaccadically; with full psychometric curves (requiring repeated presentations of a given flash luminance), this requires much more data sessions per subject. In our previous work, we reached qualitatively similar conclusions whether we used our current approach or one also including presaccadic perceptual suppression trials (Idrees et al., 2020).

### Data analysis

We only analyzed trials with button press reaction times between 300 and 3,000 ms. We also only included trials in which there were no flagged eye position samples within ± 250 ms from probe flash onset. Flagged eye position samples could occur due to blinks (missing eye position data) or eye tracker noise (e.g., by interference from eyelashes if subjects started squinting).

We detected all microsaccades using our established methods (Bellet, Bellet, Nienborg, Hafed, & Berens, 2019; Chen & Hafed, 2013). We then recalculated probe flash times relative to the recalculated microsaccade onset times (Figure 2A). This accounted for the fact that online microsaccade detection was necessarily always slightly later than actual microsaccade onset (due to the data buffering mentioned above). Trials with saccades near probe flash onset that were larger than 3 deg in radial amplitude were excluded. These were extremely rare; in fact, for all three image types, most microsaccades were less than 1 deg in amplitude (see Results).

To obtain a time course of microsaccade-related perceptual threshold elevations (immediately around microsaccades) followed by recovery (for longer latency probe flash times relative to saccade onset), we then classified all trials into three different groups according to the time of the closest microsaccade to flash onset. The first group included all trials in which the closest microsaccade to flash onset started within ± 50 ms from the probe flash event (because of our reclassification of microsaccade onset times in post hoc analyses, there could be very few trials with a flash right before a microsaccade, and that is why we included 50 ms on either side of flash onset time here; Figure 2A). This group of trials was expected to be associated with impaired detection performance (and it was called the group containing the microsaccadic suppression time bin). The second group included all trials in which the flash occurred 70 to 150 ms after the onset of the closest microsaccade to the flash. This group of trials was expected to show recovery in perceptual thresholds (and the time bin associated with it was called the recovery time bin). Finally, the third group of trials was that in which there were no microsaccades at all within ± 250 ms from probe flash onset. These trials were called the baseline trials.

We further filtered trials according to microsaccade direction. Specifically, we found that most microsaccades were predominantly horizontal (see Results), consistent with earlier observations (Engbert & Kliegl, 2003; Laubrock, Engbert, & Kliegl, 2005). Therefore, we only included trials in the analyses for which the closest microsaccade to probe flash onset (including the three temporal categorizations described above) was predominantly horizontal (directional angle within < 45 deg from the horizontal direction). This was reasonable given the vertical gratings, which would mean that horizontal microsaccades would be expected to cause the largest sensory transients in the brain after they occur (Khademi et al., 2020). Our results were largely similar when we performed the analyses across all microsaccade directions in the data, and this is consistent with our observation that most microsaccades were predominantly horizontal (see Results).

To assess perceptual performance, for each subject and time bin, we plotted the proportion of correct trials as a function of probe flash luminance increment above the background luminance. We then fit psychometric curves using the *psignifit 4 toolbox* (Schutt, Harmeling, Macke, & Wichmann, 2016); we specifically used the cumulative Gaussian function for fitting. We defined the perceptual threshold as the luminance increment of the probe flash yielding a 62.5% correct performance rate (given that ours was a four-alternative forced-choice paradigm with a 25% chance performance rate). For each subject and time bin, we estimated the threshold, and we then compared thresholds between conditions (e.g., low spatial frequency vs. white fixation spot in the suppression time bin or the suppression time bin vs. the baseline time bin) across the population by showing mean and *SEM* across all subjects.

We performed initial statistical tests using the Friedman nonparametric test. Specifically, to test for an impact of probe flash time within a given image condition, we compared thresholds across subjects by grouping the measurements into three time bins as the factors of the statistical analysis (suppression, recovery, and baseline time bins). If the Friedman test had a *p*-value of less than 0.05, we then performed pairwise Wilcoxon signed rank tests to check which factors (time bins) were associated with thresholds that were different from each other. We consider an alpha value of less than 0.05 as significant in this study. For checking whether the threshold depended on the foveated image type, we again performed a Friedman test but only on data from within a given time bin (e.g., the microsaccadic suppression time bin). This time, the factors of the statistical test were the three image types. We then used the same logic of post hoc pairwise comparisons. We report all *p*-values in Results.

We also employed a generalized linear mixed model (GLMM) to account for the fixed effects of the categorical predictor, foveated image condition, and the continuous predictors, microsaccade amplitude and probe flash time from microsaccade onset, on the response variable of interest to us, the perceptual threshold of the subjects. We did this because there could be different dependencies on factors such as microsaccade amplitude, especially since our analyses revealed systematic differences in microsaccade amplitudes across the three foveated image conditions (see Results). The GLMM model included random intercepts for each subject to account for individual differences. The model, which included 8,596 observations and was fit using maximum penalized likelihood (MPL), employed a normal distribution and an identity link function. The Akaike information criterion (AIC) was 16,251, the Bayesian information criterion (BIC) was 16,349, the log-likelihood was –8,111.3, and the deviance was 16,223. We evaluated the model fit and assumptions using diagnostic plots, with main and interaction effects reported in the Results. The model included random effects for the subjects’ identity, capturing individual variability with a standard deviation of 1.4227 for the intercept. The residual standard deviation was 0.61817.

We also assessed sensitivity at threshold by measuring the slope of the psychometric curve near the probe flash levels causing threshold performance. To do so, for each curve, we divided the difference in performance (65% minus 60% correctness rate) by the difference in luminance values, yielding a 65% and 60% correctness rate. This gave an estimate of the slope of the psychometric curve at threshold. We then performed similar statistical tests on the slope measurements as we did for the detection thresholds. However, we did not run a GLMM on the sensitivity parameter since our preliminary statistical tests suggested a lack of dependence of psychometric curve slopes on foveated visual image appearance in our data (see Results).

For visualizing microsaccade amplitudes as a function of foveated image type, we first averaged the microsaccade amplitude per condition within each subject's data. Then, we visualized the population results by averaging across subjects and showing *SEM* ranges. We also did this for microsaccade peak velocities to assess the movements’ kinematics.

We also considered the potential influences of absolute time within a trial at which the probe flashes occurred. Specifically, if microsaccade times within a given trial were tightly time-locked to image onset (at the beginning of the trial), then any perceptual fluctuations relative to microsaccade onset according to our microsaccade-contingent triggering algorithm (Figure 2A) might alternatively be explained by stimulus-induced fluctuations in perception. Therefore, we also checked the distributions of microsaccade times from trial onset (Figure 2B) and, by consequence, probe flash times from trial onset (Figure 2C). This allowed us to compare the time scales at which our flash times were presented, either relative to microsaccade onset (Figure 2A) or relative to trial onset (Figure 2C).

Finally, we characterized where subjects fixated their gaze at the time of probe flash presentation. To do so, we averaged eye position in the interval from –25 ms to +75 ms relative to probe flash time. Then, we plotted the measurements across trials for each fixated image type. We picked more time samples after the flash than before because most flashes were triggered (by design of the experiment) after a microsaccadic event (Figure 2A), and we wanted to avoid including the microsaccadic displacement itself in the eye position measurement.

## Results

We asked subjects to fix their gaze near the center of one of three possible images (Figures 1A–C). One image was a low spatial frequency vertical Gabor grating of 0.5 cycles/deg spatial frequency (Figure 1A), the other was a high spatial frequency vertical Gabor grating of 5 cycles/deg spatial frequency (Figure 1B), and the third was a small white fixation spot (Figure 1C). We then presented a brief probe flash peripherally for just one display frame (at one of the four cardinal directions; 9.1 deg eccentricity), and we asked subjects to indicate where it appeared on the display (four-alternative forced-choice paradigm) (Figures 1A–C). The probe flash was designed to appear at different time intervals relative to the occurrence of a microsaccade (Methods; examples are shown in Figures 1D–F, and full distributions are documented in Figure 2A), and the microsaccade itself was not explicitly instructed. Rather, the subjects were only told to look at the center of the image, and the computer waited for online microsaccade detection in order to trigger the probe flash (Methods).

Across all trials, in post hoc analyses, we searched for the nearest microsaccade to probe flash onset (Figure 2A). We then first confirmed that our relative timing between microsaccades and probe flash times was as expected from our experimental design. In other words, we confirmed that the times of probe flashes that we analyzed were tightly synchronized with microsaccade onset, irrespective of when microsaccades occurred within a given trial. This was expectedly the case (Figure 2A) since this is how we designed our stimulus triggering (Methods). Indeed, when we now plotted the likelihood of microsaccade onset times relative to trial onset (Figure 2B) and, similarly, the likelihood of probe flash onset times relative to trial onset (Figure 2C), we found that both of these quantities were very broadly distributed in time and not as tightly synchronized as in the distributions of Figure 2A. Moreover, there was no correlation between probe flash time relative to microsaccade onset and probe flash time relative to trial onset. Thus, any transient changes in performance that we analyzed in this study relative to microsaccade onset (on the scale of less than ∼100 ms time constants; see below) were not explained by potential time-related fluctuations in perception (VanRullen, 2016) that were time-locked to trial onset.

**Figure 3.**
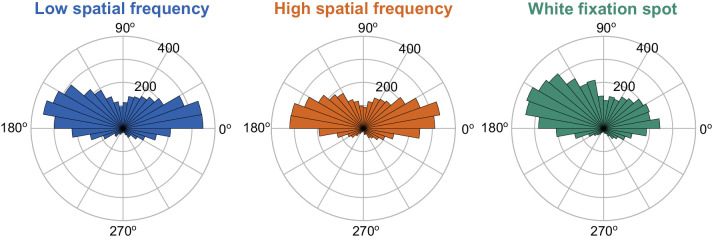
Predominantly horizontal nature of microsaccades in our experiments. Each panel shows the direction distribution of observed microsaccades (closest to probe flash onset time in every trial; Methods) for each fixated image of Figure 1 (pooled across all subjects). As can be seen, most movements were predominantly horizontal. There was also a significant paucity of downward microsaccades in all conditions.

Next, we assessed the metric and kinematic properties of the microsaccades. Independent of the underlying foveated image, most microsaccades that occurred were predominantly horizontal (Figure 3). For the grating images, this likely reflected the vertical orientation of the gratings, since orthogonal eye movements to the luminance gradient would be expected to give rise to the most useful information to the visual system about the underlying image (Rucci, Iovin, Poletti, & Santini, 2007). This is also consistent with neurophysiological signatures of microsaccade-induced visual reafferent responses at extrafoveal eccentricities, in which orthogonal eye movements scaled to a given spatial frequency give rise to the clearest modulations (Hafed et al., 2022; Khademi et al., 2020). For the white fixation spot, there were slightly more vertical eye movements than with the Gabor gratings (likely reflecting the square appearance of the fixation spot, which includes both horizontal and vertical edges); nonetheless, the overall predominantly horizontal signature of eye movement directions with the white fixation spot was consistent with previous reports (Engbert & Kliegl, 2003; Laubrock et al., 2005). All of these observations led us to focus our remaining analyses on predominantly horizontal eye movements with an absolute direction from horizontal of less than 45 deg; we obtained generally similar results when we included all trials into the analyses, as expected given the large number of predominantly horizontal movements seen in Figure 3. It is also interesting to note here that there were barely any downward microsaccades in our data at all (Figure 3); this might be related to general tendencies of the oculomotor system to bias gaze upward, whether in visual or memory conditions (Goffart, Hafed, & Krauzlis, 2012; Goffart, Quinet, Chavane, & Masson, 2006; Khademi et al., 2024; Malevich, Buonocore, & Hafed, 2020; Snodderly, 1987; White, Sparks, & Stanford, 1994; Willeke, Cardenas, Bellet, & Hafed, 2022; Zelinsky, 1996).

**Figure 4.**
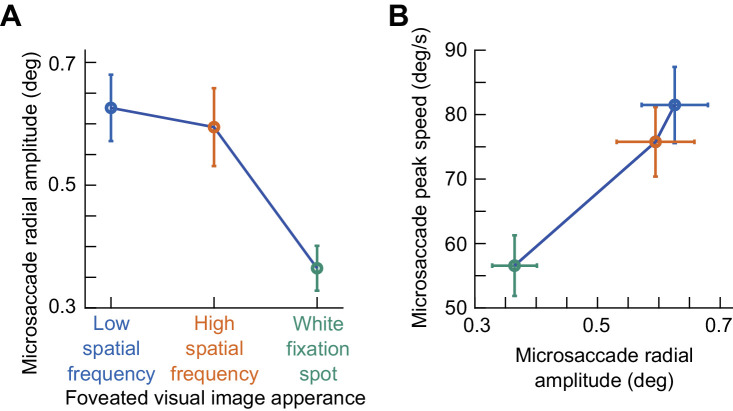
Interaction between foveated visual image appearance and microsaccade amplitudes. (**A**) For all trials with the nearest microsaccade to probe flash onset being predominantly horizontal, we plotted the radial amplitude of the microsaccade as a function of the foveated image type. Error bars denote *SEM* across subjects. Microsaccade amplitudes reflected the underlying spatial scale of the viewed foveal image, as expected, but they were always significantly smaller than 1 deg. (**B**) Same as **A** but now plotting the peak velocity of the microsaccades as a function of their amplitude. Error bars denote *SEM* across subjects. The eye movements followed the expected main sequence relationship between saccade size and saccade peak speed (Zuber et al., 1965). Also see Figure 9 for raw microsaccade amplitude distributions across image types.

In terms of movement amplitudes, we found that predominantly horizontal microsaccades tended to be slightly larger for the low spatial frequency image than for the high spatial frequency image, and microsaccades were also the smallest in size for the small white fixation spot. These results can be seen in Figure 4A, and they are consistent with the abovementioned ideas about how fixational eye movement properties can be strategically optimized by the visual-oculomotor system to maximize information gain from the underlying images. Nonetheless, in all cases, the microsaccades that we investigated in this study were always significantly smaller than 1 deg in radial amplitude regardless of the underlying image type (Figure 4A), and they also obeyed the main sequence relationship between peak velocity and amplitude (Figure 4B) (Zuber et al., 1965). Interestingly, and as we show explicitly in more detail below, we observed the strongest microsaccadic suppression for the white fixation spot condition, which had the smallest, and thus slowest, eye movements. We return to this point later in the text, after describing the subjects’ perceptual performance results in the task.

Thus, given that we have now confirmed the occurrence of small, fixational microsaccades in our experiments, we now had a situation relatively similar to that described in Idrees et al. (2020): That is, a rapid eye movement (this time, small) was generated across a textured background, and the detection of a brief probe flash away from the saccade endpoint was investigated. We now turn to describing how the detection of the probe flash varied as a function of both its time relative to microsaccade onset time as well as the underlying foveated visual image appearance. We also explore the potential influences of gaze position and microsaccade amplitude differences on the interpretation of our results.

### Both perceptual detection thresholds and sensitivity are affected in the immediate temporal vicinity of microsaccades

**Figure 5.**
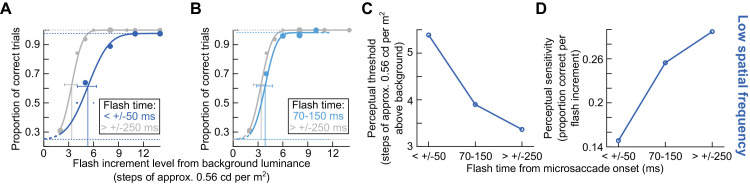
Impairment in both detection threshold and sensitivity (slope of the psychometric curve at threshold) by microsaccades in an example subject. (**A**) When viewing the low spatial frequency grating (Figure 1A), peripheral probe flashes (at approximately 9 deg eccentricity) had to be higher in luminance to be successfully detected if they occurred within ± 50 ms (blue) from microsaccade onset than if they occurred without any nearby microsaccades within ± 250 ms (gray). This figure shows the results from one example subject (S05). The continuous curves show psychometric curve fits to the shown data points (Methods), and each data point's size is scaled by the number of observations collected with the shown probe flash luminance increment above the background luminance. The vertical lines show the flash levels resulting in threshold performance (the slight y-value differences at the threshold indications reflect the slightly different asymptotic levels of performance in the two shown conditions) (Schutt et al., 2016). (**B**) Same analyses but with the flashes now occurring 70 to 150 ms after microsaccade onset. Performance recovered to near baseline performance. The gray curve is the same as that in A. (**C**) Detection thresholds of this subject as a function of flash time relative to microsaccade onset. There was clear perimicrosaccadic suppression of performance (manifested as a threshold elevation). (**D**) Similar to **C** but for measures of the slope of the psychometric curves near the threshold values (Methods). Psychometric curves were shallower for probe flashes within ± 50 ms from microsaccade onset.

Figure 5 shows psychometric curves characterizing the performance of one example subject (S05) when fixating the low spatial frequency grating. The baseline curve (gray in Figures 5A, B) was obtained from all trials in which there were no microsaccades occurring within ± 250 ms from probe flash onset (Methods). For the other shown curves, a predominantly horizontal microsaccade started either within ± 50 ms from probe flash onset (i.e., during the expected microsaccadic suppression time bin; Figure 5A) or 70 to 150 ms before probe flash onset (i.e., during a recovery time bin with the microsaccadic event being sufficiently far away in time; Figure 5B). As can be seen, the subject's performance in the task was clearly impaired in the microsaccadic suppression time bin, as evidenced by the lower proportion of correct trials in every flash level that was neither too difficult (floor effect) nor too easy (ceiling effect). For probe flashes longer after a microsaccade (in the recovery time bin), performance recovered and approached that observed in the baseline trials (Figure 5B). Such time course of microsaccadic suppression followed by recovery is directly consistent with many earlier studies of saccadic suppression (Beeler, 1967; Hafed & Krauzlis, 2010; Ibbotson & Krekelberg, 2011; Zuber & Stark, 1966), and it is, therefore, also consistent with our interpretation of Figure 2 above that our results cannot be explained solely by long-term fluctuations in perception that may or may not be independent of the eye movements (Bellet et al., 2017; VanRullen, 2016).

The impairment of the example subject's performance in the microsaccadic suppression time bin was manifested in two ways. First, there was an increase in detection threshold. For example, during the microsaccadic suppression time bin (Figure 5A), the probe flash needed to be almost approximately 3.1 cd/m^2^ brighter than the background to result in a 62.5% correctness rate in task performance (Figure 5C, leftmost data point). This was a Weber contrast value of 0.14. On the other hand, during baseline, the probe flash needed to be only approximately 1.9 cd/m^2^ brighter than the background luminance (Figure 5C, rightmost data point), equivalent to a 0.09 Weber contrast. Second, the sensitivity of performance to subtle luminance changes of the probe flash was also impaired. This is evidenced by the shallower slope of the psychometric curve of the subject during the microsaccadic suppression time bin (Figure 5A) when compared to the recovery and baseline conditions (also quantified in Figure 5D). In the recovery time bin (Figure 5B), the slope of the psychometric curve was more similar to that in baseline, suggesting an expected gradual return to baseline sensitivity with time (Figure 5D). Thus, both the detection threshold and sensitivity (slope of the psychometric curve at the perceptual threshold) of the subject were impaired in association with microsaccades.

**Figure 6.**
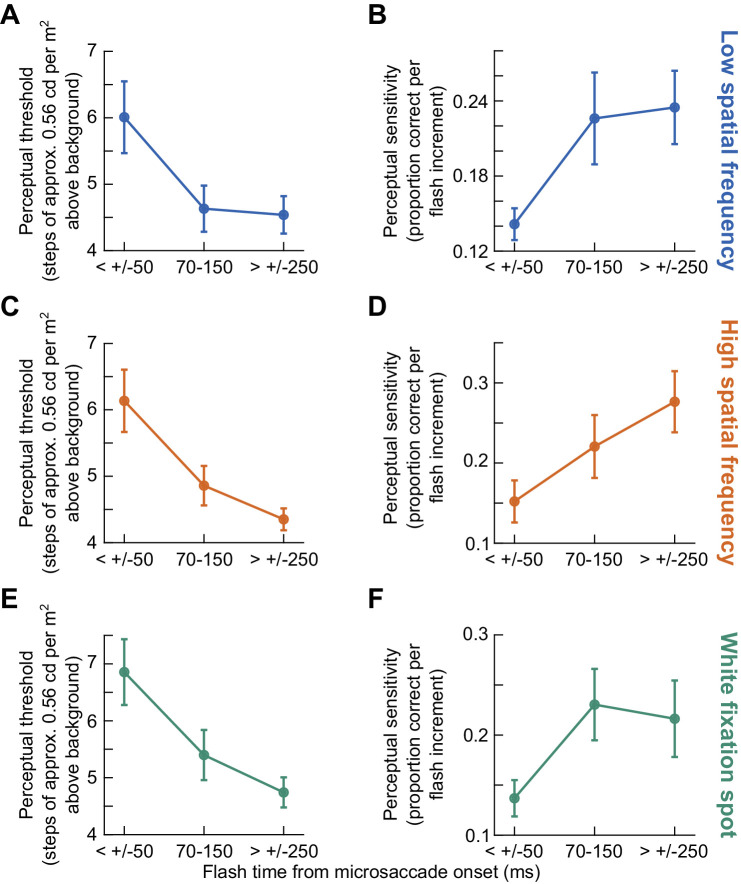
Summary across subjects. (**A**) Detection thresholds as a function of flash time from microsaccade onset when viewing the low spatial frequency grating. This figure is similar to Figure 5C but averaging across all subjects. Error bars denote *SEM* across subjects. There was a threshold elevation near microsaccade onset and recovery for larger temporal separation between microsaccades and flash times. (**B**) Same as **A** but for the slopes of the psychometric curves when viewing a low spatial frequency grating. This figure is thus similar to Figure 5D. (**C**, **D**) Same as **A** and **B** but for the case of viewing the high spatial frequency grating. Qualitatively similar observations were made. (**E**, **F**) Same as **A** and **B** but for the case of viewing the white fixation spot. Again, qualitatively similar observations were made. See Figure 7 for quantitative comparisons.

Across all subjects, microsaccadic suppression affected both detection thresholds and sensitivity (psychometric curve slopes) and for all foveated visual image appearances that we tested. This is best seen by the analyses of Figure 6. Here, we plotted in the left column (Figures 6A, C, E) the detection thresholds of all subjects as a function of probe flash time relative to microsaccade onset time. The different panels denote the different fixated images, and the error bars denote *SEM* across subjects. In each panel, there was an elevation of perceptual detection thresholds in the microsaccadic suppression time bin, which recovered in other time windows. As for sensitivity (the slope of the psychometric curve at the perceptual threshold flash level), the results are shown in Figures 6B, D, F. Here, the microsaccadic suppression time bin was associated with generally reduced sensitivity (shallower psychometric curves) relative to the other two analyzed time windows, and this happened for all foveated image types. Statistically, these results were robust. Specifically, within each image type, there was a significant effect of flash time on perceptual thresholds (*p* = 0.0061992, 0.0001872, and 0.00030864 for the low spatial frequency, high spatial frequency, and white fixation spot, respectively; Friedman test with time bin as factor). There were also effects on the slopes of the psychometric curves (*p* = 0.024611, 0.00050886, and 0.022274 for the low spatial frequency, high spatial frequency, and white fixation spot; Friedman test with time bin as factor). In post hoc comparisons, the threshold in the microsaccadic suppression time bin was systematically different from that in the baseline time bin (*p* = 0.0073, 0.0005, and 0.0005 for the low spatial frequency, high spatial frequency, and white fixation spot, respectively; Wilcoxon signed-rank test; also see below for further tests based on GLMMs). Similarly, the slopes tended to also be different between the microsaccadic suppression and baseline time bins (*p* = 0.0049, 0.0063, and 0.04 for the low spatial frequency, high spatial frequency, and white fixation spot, respectively; Wilcoxon signed-rank test). Thus, both perceptual detection thresholds and sensitivity (psychometric curve slopes) were affected in the immediate temporal vicinity of microsaccades.

### Only perceptual detection thresholds might depend on the foveated image appearance

**Figure 7.**
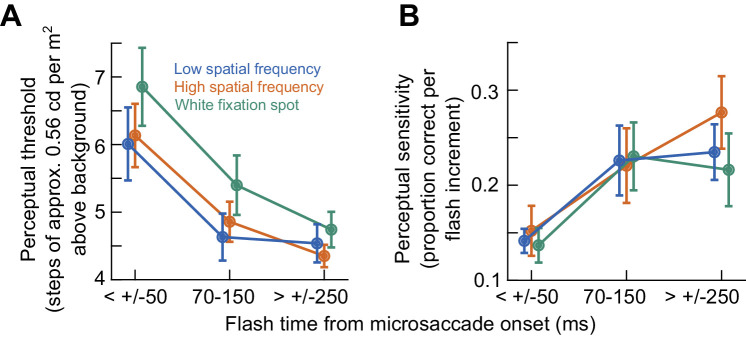
Stronger microsaccadic suppression for the small white fixation spot. (**A**) We plotted the threshold values of Figure 6 together in one graph. Subjects exhibited higher detection thresholds for the white fixation spot, suggesting stronger microsaccadic suppression. Moreover, this stronger suppression persisted in the recovery time bin (70–150 ms from microsaccade onset), and it was only in baseline that the thresholds for all three viewed image types were statistically similar. Quantitatively, the thresholds during the microsaccadic suppression time bin were 3.84, 3.44, and 3.36 cd/m^2^, respectively, for the white fixation spot, high spatial frequency, and low spatial frequency grating (equivalent to 0.173, 0.155, and 0.152 Weber contrast, respectively). (**B**) For the slopes of the psychometric curves, there were no differences across viewed image types in any of the time bins. Thus, only detection thresholds showed an image dependence of microsaccadic suppression in our data.

Despite the qualitatively similar results in Figure 6 across all three foveated visual image appearances, when we quantitatively compared these results, we found that microsaccadic suppression of peripheral perceptual detection performance was strongest when viewing the small white fixation spot rather than when viewing a low spatial frequency grating, as we had previously observed with large saccades (Idrees et al., 2020). Consider, for example, Figure 7A, which combines the threshold plots of Figure 6 together into a single visualization. In the microsaccadic suppression time bin, there was a higher threshold value for the white fixation spot than for the low spatial frequency grating (*p* = 0.01389; Friedman test comparing all three image conditions; and *p* = 0.0161; posthoc Wilcoxon signed-rank test comparing the white fixation spot to the low spatial frequency grating condition). This effect also continued in the recovery time bin (*p* = 0.0022806; Friedman test; *p* = 0.002; post hoc Wilcoxon signed-rank text comparing the white fixation spot to the low spatial frequency grating condition), consistent with the higher threshold for the white fixation spot in the microsaccadic suppression time bin. In the baseline time bin, all the detection thresholds were statistically similar to each other (*p* = 0.35255; Friedman test comparing all three image conditions in the baseline time bin). During microsaccadic suppression, the high spatial frequency performance was intermediate between the two and closer to the low spatial frequency condition. Thus, in terms of detection thresholds, perimicrosaccadic suppression of peripheral perceptual detection performance was strongest for the white fixation spot, as opposed to either a low or high spatial frequency grating.

Figure 7A also suggested a trend for a higher threshold in the white fixation spot condition than in the other two image conditions even in the baseline time bin. This could reflect the stronger microsaccadic suppression effect in the earlier time bins. For example, with larger saccades, conditions causing stronger saccadic suppression also have longer recovery time courses (Baumann et al., 2021; Idrees et al., 2020). However, 250 ms between microsaccade and probe flash onset (which was our minimal temporal separation for the baseline trials) is a long time, and perceptual recovery from both microsaccades and saccades usually happens much faster than this (e.g., Figure 6). Thus, a more likely explanation is that the overall microsaccade rates were higher in the white fixation spot condition than in the Gabor grating conditions. This is expected given the punctate nature of the fixation spot (Poletti & Rucci, 2010), and we also confirmed it in our data: Median inter-microsaccadic intervals were 395 ms for the white fixation spot condition, whereas they were 440 ms and 453 ms for the low and high spatial frequency grating conditions, respectively. Thus, in the baseline time bin of the white fixation spot condition, it was more likely that the probe flashes could happen closer to other microsaccades, causing slightly elevated thresholds (on average) than in the grating conditions.

Importantly, the above observation, as well as the recognition that there could be multiple factors influencing our threshold results beyond just the foveated visual image appearance, prompted us to explore interactions between various experimental parameters in more detail. We used a GLMM (Methods), and we specifically fit the model to analyze the impacts on perceptual thresholds of the foveated visual image condition, microsaccade amplitudes (Figure 4), and probe flash times from microsaccade onsets, as well as their interactions (Methods). The intercept in the model, representing our reference condition, the low spatial frequency grating foveated image, was estimated at β_0_ = 5.4972 (*SE* = 0.41087, *p* < 10^−40^), indicating the expected visual perceptual threshold when all other predictors were at their reference levels. Further analysis of the model parameters revealed significant fixed effects for probe flash times from microsaccade onset (β_a_ = –0.60076, *p* = 0), indicating a substantial decrease in the response variable (perceptual threshold) with increasing probe flash times relative to microsaccade onset. This result shows that saccadic suppression was indeed present in our data (Figure 7A) and that it was strongest closest to microsaccade onset. Microsaccade amplitude also had a significant negative effect (β_b_ = –0.028036, *p* = 0.01331). The high spatial frequency grating and white fixation spot both had significant positive effects (β_c_ = 0.15397, *p* < 10^−20^ and β_c_ = 0.66634, *p* < 10^−253^, respectively), with the fixation spot having the larger coefficient value. By definition of the model, these coefficients were relative to the low spatial frequency grating as the reference. They thus afford us some additional interpretations. For example, given the large difference in coefficient size for the microsaccade amplitudes compared to the image conditions, we are confident that the eye movement amplitudes had negligible impacts on the results of Figure 7A. Interestingly, though, there was an interaction between the high spatial frequency grating condition and microsaccade amplitudes (β_bc_ = 0.12688, *p* < 10^−15^), suggesting that the effect of microsaccade amplitude varied depending on the image condition. With a high spatial frequency grating, changing the microsaccade size could substantially change the spatiotemporal profile of the retinal image shift (see Discussion), so we indeed expected such an interaction to occur. In line with this thought, there was no significant interaction between microsaccade amplitude and the white fix spot condition. Several other interaction terms were also significant, such as the interaction between probe flash time from microsaccade onset and the high spatial frequency grating (β_ab_ = 0.089773, *p* < 10^−8^). All results, suggesting a nuanced interplay between predictors and significant variability across subjects, are summarized in Table 1.

**Table 1.** Fixed effects and interaction terms in the GLMM. This table presents the statistical output for each predictor and interaction term in the GLMM. We used the model to evaluate the impacts of image type, microsaccade amplitude, and probe flash time from microsaccade onset on the perceptual thresholds of our subjects.

Predictor	Coefficient (β)	*SE*	tStat	*p*-value
Low spatial frequency grating (intercept)	5.4972	0.41087	13.379	2.03 × 10^−36^
Probe flash time from microsaccade onset	−0.60076	0.011754	−51.111	0
Microsaccade amplitude	−0.028036	0.011324	−2.4759	0.01331
High spatial frequency grating	0.15397	0.016477	9.3446	1.16 × 10^−16^
White fix spot	0.66634	0.018951	35.161	3.88 × 10^−249^
Probe flash time from microsaccade onset * Microsaccade amplitude	0.002953	0.010759	0.27446	0.78374
Probe flash time from microsaccade onset * High spatial frequency grating	0.089773	0.016644	5.3937	7.09 × 10^−04^
Probe flash time from microsaccade onset * White fix spot	0.055707	0.017923	3.1081	0.0018891
Microsaccade amplitude * High spatial frequency grating	0.12688	0.015797	8.032	1.09 × 10^−11^
Microsaccade amplitude * White fix spot	−0.025915	0.019699	−1.3155	0.18836
Probe flash time from microsaccade onset * Microsaccade amplitude * High spatial frequency grating	−0.053843	0.015502	−3.4734	0.0005165
Probe flash time from microsaccade onset * Microsaccade amplitude * White fix spot	0.10285	0.018374	5.5975	2.24 × 10^−04^

Interestingly, unlike the thresholds, the slopes of the psychometric curves of the same subjects did not appear to depend on the foveated visual image appearance during the microsaccadic suppression time bin (*p* = 0.59156; Friedman test comparing all three image conditions). This can be seen in Figure 7B. For all image types, the shallower slope during the microsaccadic suppression time bin was similar in value. This was also the case for the higher slopes seen in the recovery and baseline time bins. Thus, it was only the detection thresholds, and not the slopes of the psychometric curves, that showed a potential image dependence of microsaccadic suppression of peripheral perceptual detection performance (and that is why we did not run a GLMM for the slopes of the psychometric curves). It would be interesting in future studies to investigate why this was the case.

We next considered whether the results of Figure 7A and Table 1 could be explained by factors other than microsaccadic suppression per se, especially ones that we did not include in our GLMM above. Specifically, since there was no specific marker to fixate on in the grating images, it could be the case that the subjects were biased in where they directed their gaze during the trials with grating images. For example, if these subjects systematically fixated their gaze slightly upward relative to the grating center, then this could have rendered one flash location (the upper one in this example) significantly closer to the gaze center than in the case of the small white fixation spot, with much more focused gaze direction. This would have made one flash location easier to detect than with the white fixation spot. However, this logic fails since a bias in gaze position with the gratings toward one probe flash location would render the three other flashes actually farther away from the gaze center and therefore harder to detect. If anything, this should have made the overall task harder with the grating images than with the white fixation spot. This was clearly not the case in our data (subjects performed worse during the microsaccadic suppression time bin with the white fixation spot). We also have three additional reasons to rule out a potential influence of gaze position (and thus probe flash visibility) on the results of Figure 7A.

**Figure 8.**
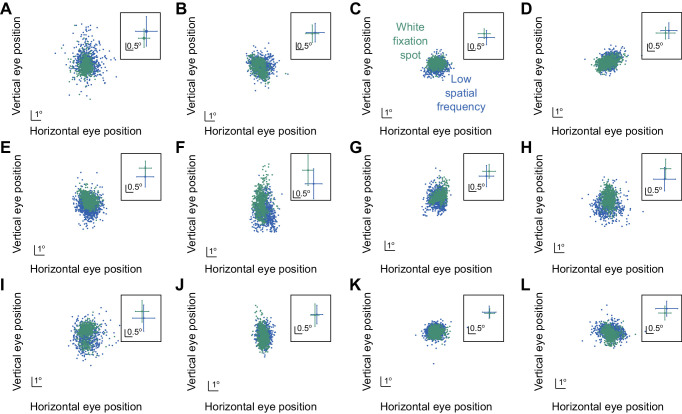
Lack of large systematic gaze position biases with grating images. For each subject (**A**–**L**), we measured eye position at the time of probe flash presentation (Methods). In each panel, each dot represents a single trial, and the different colors indicate which image was being viewed by the subject (the color legend in **C** applies to all panels). The inset in each panel shows the mean and standard deviation values of the corresponding raw data plots of the panel, and **E** contains the eye positions of the same example subject whose psychometric curves were shown in Figure 5 (S05). As can be seen, all subjects fixed their gaze at very similar positions between the two image types. There was clearly larger dispersion of fixation position with the grating images (due to a lack of a specific punctate marker), but this dispersion was largely symmetric in all directions. Thus, all four peripheral flashes were, on average, at a similar retinotopic eccentricity when they appeared, ruling out a simple retinotopic visibility as the primary explanation of the results of Figure 7A.

First, we explicitly measured gaze position at the time of probe flash presentation across all trials and image types (Methods). Figure 8 shows these measurements for each subject individually, with the blue dots showing trials with the low spatial frequency grating and green dots showing trials with the white fixation spot. We did not plot the high spatial frequency grating data in order to reduce clutter in the figure, but these data were virtually identical to those of the low spatial frequency grating data (consistent with Figure 4). The insets show the mean and standard deviations of the shown raw data points. As can be seen, while it was certainly true that gaze position was more dispersed with the grating images, as expected, the subjects correctly followed our instructions to maintain their gaze near the center of the image (average gaze position was similar whether the subjects were viewing a grating or a small white fixation). Quantitatively, deviations in mean gaze position between the white fixation spot and the grating cases were always smaller than approximately 0.5 deg and often significantly smaller. Given that our peripheral probes were at 9.1 deg, this small difference in average gaze position was not expected to influence detectability. In fact, even at 5 deg, we found in an earlier study that such gaze position deviations of the same magnitude as those observed here did not alter peripheral detection performance (Bellet et al., 2017).

Second, in the baseline time bin in Figure 7A, perceptual performance was similar for all image types (despite the slight tendency of an elevation for the white fixation spot, which we discussed above). Thus, if gaze position was indeed systematically biased for the gratings relative to the white fixation spot, then we should have also seen a difference in performance during the baseline time bin. This was not the case.

Third, the stronger microsaccadic suppression of peripheral detection performance for the white fixation spot (as opposed to the grating images) was specific for threshold values but not for the slopes of the psychometric curves (Figure 7B). If gaze position altered the visibility of the peripheral targets between the different image types, then we might have expected similar changes in both thresholds and sensitivity across image types. Therefore, all of these observations, coupled with the results of Figure 8, suggest that systematic gaze position differences across image conditions likely do not fully explain the results of Figure 7.

**Figure 9.**
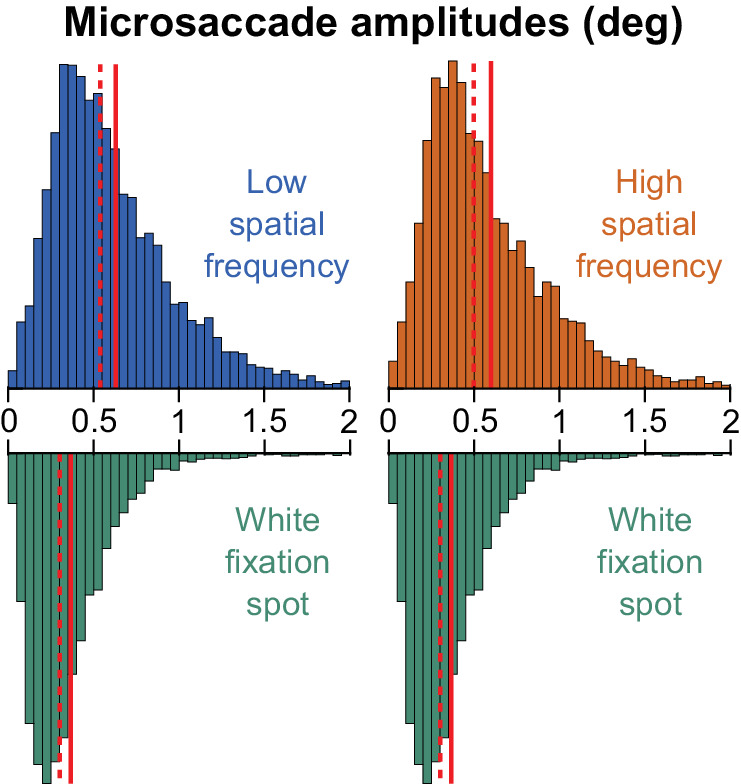
Microsaccade amplitude distributions. In this figure, we plotted the raw microsaccade amplitude distributions underlying the summary statistics in Figure 4. We placed the distribution for the white fixation spot under each of the low or high spatial frequency gratings for easier comparison. In all cases, most microsaccades were smaller than 1 deg in amplitude. Thus, there was large overlap across conditions. In each distribution, the dashed vertical line indicates the median, and the solid vertical line indicates the mean.

This leaves a final question related to microsaccade size. While we already ruled this out with our GLMM analyses described above, we document additional observations on microsaccade size in even more detail here for completeness, especially noting the influence of microsaccadic peak velocities. In particular, smaller microsaccades are associated with lower peak velocities (Figure 4), and we observed that microsaccades were slower, on average, with the white fixation spot when compared to the grating images. However, slower movements should cause milder image transients and blurs than faster movements, which should, in principle, be associated with milder saccadic suppression. This is opposite to what we observed experimentally, with stronger microsaccadic suppression for the white fixation spot condition. Moreover, there seems to be a dissociation between saccade speed and saccadic suppression strength in general (Gremmler & Lappe, 2017). And, an early study with large saccades actually documented larger saccadic suppression effects with larger (and faster) saccades (Mitrani, Yakimoff, & Mateeff, 1970), again opposite of what we observed. These ideas provide further support for our interpretation that microsaccade amplitude (and peak velocity) had a minor influence on our observations. Nonetheless, we investigated our microsaccade amplitude distributions more closely. Despite the differences in average microsaccade sizes that we observed in Figure 4, there was a large overlap in the raw distributions of microsaccade sizes, as can be seen from Figure 9 (with most microsaccades being smaller than 1 deg in all conditions). Therefore, there was also a large overlap in speed distributions. Thus, it does not seem likely that the results of Figure 7A could be fully accounted for by microsaccade size or speed.

Therefore, our analyses, combined, suggest that microsaccadic suppression in our experiments did occur for all tested visual image types at the fovea and that the suppression was stronger when the viewed foveal visual image was a small white fixation spot as opposed to either a low or high spatial frequency texture.

## Discussion

In this study, we investigated the dependence of perimicrosaccadic suppression of peripheral perceptual detection performance on the visual appearance of the images across which microsaccades were generated. This was a microsaccadic correlate of studies in which saccadic suppression was researched, with larger saccades being made across textured backgrounds (Idrees et al., 2020). Unlike a potential expectation from these studies, we did not find stronger microsaccadic suppression for the low spatial frequency grating. Rather, the strongest suppression occurred when microsaccades were made across a small, foveal fixation spot (Figure 7A). Moreover, only detection threshold elevations depended on the foveal visual image appearance but not the slope reductions of the psychometric curves.

There have been previous investigations relating microsaccadic suppression to the visual properties of the scene, particularly for the case of spatial frequency (Chen & Hafed, 2017; Hass & Horwitz, 2011; Intoy et al., 2021; Scholes et al., 2018). Using monkeys, Hass and Horwitz (Hass & Horwitz, 2011) investigated the behavioral detection of peripheral grating stimuli, and they found impaired detection in the presence of microsaccades. They also recorded from neurons in the primary visual cortex of the monkeys, and they found evidence of microsaccadic suppression. Such evidence was also found in the superior colliculus of monkeys (Chen & Hafed, 2017), and that study again also demonstrated a behavioral correlate of microsaccadic suppression in the performance of the animals. However, unlike in our current investigation, both of these previous monkey studies had the grating being presented peripherally. In our current case, the grating stimulus was foveal, and the detection stimulus was peripheral (and without any other background images). Thus, in this regard, our current experiments were also different from those of Scholes et al. (2018), in which both the underlying spatial frequency image and the detection stimuli were foveal. This was also the case for another study investigating foveal microsaccadic suppression of perceptual performance (Intoy et al., 2021).

Our effect on threshold elevations, being slightly larger for the small white fixation spot than for gratings (Figure 7A), was quite different from what we observed earlier with much larger saccades (Idrees et al., 2020), in which there was stronger saccadic suppression for low than high spatial frequency background textures. This difference in results might be a manifestation of the significantly slower and smaller eye movements studied here when compared to our earlier experiments. For example, with the small eye movements, shifting gaze on the low spatial frequency grating might be more similar to shifting gaze over a blank, given how gradually the grating luminance changes with the diminutive angular displacements associated with microsaccades. Similarly, with the high spatial frequency grating, the image displacement caused by the average microsaccade size that we observed (approximately two thirds of a degree; Figure 4) might have shifted the image by a whole-number multiple of luminance cycles (e.g., three cycles), just averaging the luminance modulations across the cycles out by the time the eye movement was finished. On the other hand, with the broadband fixation spot, shifting gaze over it would be expected to influence multiple spatial frequency visual processing channels and might cause stronger saccadic suppression. This interpretation is consistent with the idea that saccades of different sizes have different spatiotemporal profiles of retinal image modulations when they occur (Mostofi et al., 2020). This interpretation is also consistent with the idea that saccades in general can sculpt visual responses in the primary visual cortex (MacEvoy et al., 2008), superior colliculus (Khademi et al., 2020), and other visual brain areas when they occur. Thus, the interactions between background image spatial frequency content and the strength of saccadic suppression (Idrees et al., 2020) should not always be identical for different saccade sizes; rather, these interactions might reflect the specific sensory consequences of the particular saccades being generated.

Given that microsaccades to a small spot can overshoot it slightly (Tian et al., 2016, 2018; Willeke et al., 2022; Willeke et al., 2019), making a microsaccade in our white fixation spot condition was additionally equivalent to crossing a luminance edge (e.g., the preferred retinal locus went from a gray background to being over a white image patch and then to being over a gray background again by the end of a given microaccade). This is similar to our recent observation that when large saccades crossed a luminance bar, we observed stronger saccadic suppression than when the saccades were made across a blank (Baumann et al., 2021). Thus, a second potential explanation of the results of Figure 7A is that the microsaccades with the white fixation spot were crossing a luminance discontinuity. This would still be consistent with a visual component to microsaccadic suppression, as with larger saccades. However, in the current study, the receptive fields experiencing the peripheral probe flashes (especially if they were small in early visual areas like retina, lateral geniculate nucleus, and primary visual cortex) likely never crossed luminance bars when the microsaccades happened with the white fixation spot; these receptive fields presumably always experienced a gray background since the probe flashes were at a peripheral eccentricity. Thus, it is not clear whether in our earlier study (Baumann et al., 2021), it was the foveal crossing or the crossing of the receptive fields seeing the probes of a luminance bar that ultimately caused the stronger saccadic suppression. It would be interesting in the future to investigate this issue further.

Indeed, all descriptions above include an implicit assumption that the visual conditions in the fovea in our current experiments could influence peripheral performance even though the peripheral probe flashes themselves occurred over a completely gray background. However, this is not the first time that probes over a gray background were shown to be affected by visual conditions far from them. For example, in some experiments in Idrees et al. (2020), we had probe flashes over a gray background (with the same retinal locus being stimulated by gray both before and after saccades), with only the far surround having different textures. We still obtained altered saccadic suppression strengths with the differing far surrounds. Thus, it is still possible that our results in Figure 7A could be affected by the foveal image even though the probes were peripheral. Indeed, the thresholds that we observed in the current study during the microsaccadic suppression time bin (e.g., 0.173 Weber contrast for the white fixation spot) were slightly higher than those observed in Baumann et al. (2021) with saccades made across a completely blank background in the same experimental setup (0.13 Weber contrast). Thus, it could be the case that the foveal visual conditions in the current study could still influence peripheral performance over a gray background.

This leads to the intriguing question of how and why peripheral sensitivity can be impaired so much when tiny microsaccades occur. In the above example, our perceptual thresholds with the white fixation spot were slightly higher than those we observed earlier with much larger saccades over a gray background (Baumann et al., 2021). This also happens at the neuronal level, at least at the level of the superior colliculus, a structure relevant for saccadic suppression (Berman et al., 2017; Lee et al., 2007; Phongphanphanee et al., 2011): Even very eccentric collicular receptive fields (preferring > 20 deg of eccentricity) experience massive suppression of visual neural sensitivity to probe onsets whenever tiny microsaccades occur (Chen & Hafed, 2017; Hafed, Chen, & Tian, 2015; Hafed & Krauzlis, 2010). Of course, one aspect of this could still be visual. For example, in the superior colliculus, eccentric receptive fields can be very large. Thus, moving them by microsaccadic amounts can still cause luminance transients associated with the display edge moving on the retina relative to the dark surroundings of the display region. That is, a single peripheral receptive field (e.g., in the superior colliculus) can still experience both the display and the dark background within it and thus be exposed to a visual edge movement whenever tiny microsaccades occur. This idea implies that visual brain areas with small receptive fields should experience weaker microsaccadic suppression than in the superior colliculus.

There could also be other nonvisual components for such a disparate difference between saccade size and the peripheral eccentricity that experiences suppression. For example, if microsaccade-related motor bursts in the superior colliculus were to vary as a function of the image appearance, as is the case with larger saccades (Baumann et al., 2023), then a dependence of microsaccadic suppression on foveal visual image appearance could emerge peripherally through extraretinal mechanisms (with concepts like corollary discharge). It seems likely that microsaccade-related superior colliculus motor bursts would exhibit image dependence given the current evidence in the literature so far. For example, these motor bursts can disappear completely for microsaccades made toward a blank (Willeke et al., 2019), just like with larger saccades (Baumann et al., 2023; Edelman & Goldberg, 2001; Mohler & Wurtz, 1976; Zhang et al., 2022). However, an explicit experiment probing collicular microsaccade-related motor discharge with different underlying foveal textures is warranted.

Another nonvisual component of our results could relate to the long-standing question of voluntary control over microsaccades (Willeke et al., 2019) and whether different levels of endogenous control over saccade generation (in general) could affect the strength of saccadic suppression (Gremmler & Lappe, 2017). Interestingly, Gremmler and Lappe (Gremmler & Lappe, 2017) found that saccadic suppression is weaker with exogenously driven, reactive saccades (triggered by the sudden appearance of a peripheral stimulus) than with preplanned saccades (generated endogenously based on a previous instruction to wait for a go signal). In this case, we may interpret our fixation spot condition as invoking a higher level of endogenous control over microsaccade generation than the two grating image conditions. This is not unreasonable. For example, in tasks with a small fixation spot, we found that microsaccades optimize eye position by reducing foveal motor error (Bellet et al., 2017; Tian et al., 2016, Tian et al., 2018). This purposeful nature of the eye movements was eliminated in our two grating image conditions, and this could be yet another explanation for our elevated thresholds in the white fixation spot condition. Indeed, we found higher microsaccade rates in the fixation spot condition.

We would also like, in future experiments, to consider the visual field locations of probe flashes. In our current study, we did not separate the analyses by probe flash location because this would have required much more collected data. However, we do know from our recent perceptual and neurophysiological experiments that saccadic suppression, whether for large saccades or microsaccades, is weaker in the upper retinotopic visual field (Fracasso, Buonocore, & Hafed, 2023). We can, therefore, use these observations and combine them with the current ones to potentially explore interactions between visual field locations and underlying foveated images.

Finally, it would be interesting in follow-up experiments to present our probe flashes more centrally, such that they still appear on the low or high spatial frequency gratings themselves and not over the gray background. In that case, we can expect higher overall detection thresholds than with a blank background (Baumann et al., 2021), but it remains to be seen whether microsaccadic suppression of performance would now be stronger for the low spatial frequency grating than for the high spatial frequency grating. In such experiments, one can even parametrically change grating size relative to probe flash location to find the extent of overlap between background images and probe flashes that is needed to result in an image dependence of microsaccadic/saccadic suppression. This can, in turn, allow predicting the effective sizes of receptive fields that would be most relevant for the visual component of saccadic and microsaccadic suppression (e.g., in retina, lateral geniculate nucleus, primary visual cortex, superior colliculus, or elsewhere). This would also complement previous original perceptual experiments, in which microsaccadic suppression was indeed investigated while human participants foveated images of different spatial frequencies (Scholes et al., 2018).

Overall, we believe that our results demonstrate the relevance of studying saccadic suppression using different eye movement sizes and directions and also the relevance of considering both visual and motor components of this highly ubiquitous and robust perceptual phenomenon.
